# Pedagogic Interest Group: a novel and proven collaborative, adhocracy research group structure

**DOI:** 10.15694/mep.2021.000061.1

**Published:** 2021-03-02

**Authors:** Andrew Martin Lunn, Andrea Manfrin

**Affiliations:** 1 University of Central Lancashire

**Keywords:** Education, Management, Pedagogy, Research, Strategy, Structure

## Abstract

This article was migrated. The article was not marked as recommended.

**Background:** Teaching is a core activity for universities, and pedagogic research is essential for improving student experience, staff satisfaction, and REF and TEF scores. Pedagogic research is often performed as a secondary research area or by part-time staff, requiring good collaboration. Existing research structures in universities often result in pedagogic research falling through the gaps and for quality work and pedagogic improvements to be missed.

**Aim:** To develop a clear and flexible structure to improve participation in and the output of pedagogic research in the School of Pharmacy and Biomedical Sciences at the University of Central Lancashire.

**Methods:** A collaborative adhocracy called the Pedagogic Interest Group (PIG) was created in January 2020. It was designed to allow collaborative, flexible research projects to be easily set up by any member of staff. The group supervises and organises a bespoke team of people for each project, drawing on all previously involved staff’s expertise and contacts through an initial project meeting organised by an independent group chair. Each project group runs independently, with further help available from the group chairs.

**Results:** Under the PIG structure, seven projects have been undertaken in less than one year, with two papers published, one under review, two in preparation, one abstract accepted at an international conference, fifteen funded undergraduate research projects completed. Part-time teaching staff are more involved in the research. Internally, three departments and externally, three other UK universities have been collaboratively involved in research projects.

**Conclusion:** The PIG structure works and depends on staff’s continued engagement and at least two independent chairs for impartiality and transparency.

## Introduction

### Background

Pedagogic research represents the theoretical and/or conceptual understanding of teaching and learning processes, experiences and outcomes. It is conducted in many university departments and is a critical part of academic life, informing and improving the university’s core function of teaching. Simultaneously, it contributes to the research output of a department. Thus, pedagogical research can contribute significantly to the Research Excellence Framework (REF) and Teaching Excellence Framework (TEF) scores, in parallel to improving teaching and student experience ( Swing and Ross, 2016; Gunn, 2018; Morris, 2020).

Pedagogic research is often not the primary research focus of academics or is conducted by part-time teacher practitioners. This is the case in the School of Pharmacy and Biomedical Sciences at the University of Central Lancashire (UCLan); therefore, a new pedagogical research structure was devised and implemented, called the Pedagogic Interest Group (PIG).

### Traditional research structure(s)

The traditional research structure at a University is organised in a subject-specific hierarchical manner, with individual researchers pursuing their area of expertise. Looking at the structure in a top-down approach, the university completes a REF return, dividing funding up between faculties and departments. Each department of the university will then be further organised, potentially by discipline, with researchers working on their areas of expertise and hopefully collaborating within the department, university or broader research context, possibly in research communities ( Chirikov, 2013; Ng and Pemberton, 2013; Webber and Calderon, 2015). However, the individual researchers are responsible for securing further funding and producing discipline-specific research outputs for the university’s REF return, which is at odds with a collaborative culture ( Woiwode and Froese, 2020). Some structures, such as centres for excellence, have shown promise in increasing collaborative research between multiple disciplines ( Borlaug and Langfeldt, 2019). Nevertheless, there is often no clear structure or guidance on achieving a successful and collaborative research strategy, which can lead to bias in who collaborates, favouring those with pre-existing connections ( Birnholtz, 2007; Holman and Morandin, 2019).

### Pedagogic context

When this research landscape is applied to pedagogical research, particularly in a professional discipline such as pharmacy, medicine or nursing etc., further challenges are faced, for example, conducting research jointly within the governance of a university and hospital ( Engbers *et al.*, 2013; Cotton, Miller and Kneale, 2018). The research must also fulfil TEF criteria. Indeed, much departmental staff will be teaching focussed, employed predominantly as experts in their field with little experience in producing research outputs (e.g. teacher practitioners) ( Laird, 2012). In clinical disciplines, some of the teachers may even increasingly be patients, as experts by experience and have no academic background at all ( Lunn *et al.*, 2020). For those who are unsure where to start, what constitutes a worthwhile research study or who struggle to find the time, a lack of institutional pedagogical research structure is a major barrier to entry ( Stierer and Antoniou, 2004; Cotton, Miller and Kneale, 2018).

### Research-Based Communities of Practice

Lai and Pemberton ( Ng and Pemberton, 2013) suggested that research is an integral part of higher education institutions’ work and looked at the value of membership in communities of practice in higher education and the potential impact on subsequent research. Their research focused on:

• what individuals gain from their membership;
• how such membership enhances their research;
• where and how communities of practice can integrate within higher education, and the role they play in developing research outputs.

Communities of practice are formed naturally and informally, bound together by shared expertise ( Wenger and Snyder, 2000). Some examples of communities of practice are clinical practice, business management and information communication.

Lai and Pemberton ( Ng and Pemberton, 2013) identified 20 values of communities of practice, which are summarised in Table 1.

**Table 1:** The 20 values of research-based communities of practice in higher education, Lai and Pemberton ( Ng and Pemberton, 2013)

Autonomy and freedom to think	Alternative perspective and cross-pollination of ideas *	Time and energy-saving *	Networking, information sharing and updates
Multiple sources of ideas	Overcoming intellectual isolation *	An informal ground for learning and training	Support and guidance
Sounding board	Move towards collaborative research	Fostering of tangible returns *	Sense of belonging
Intellectual discussion	How you respond to research to pressure	Driving research *	Having a distinct identity
Like-mindedness (shared values)	Synergy and leverage *	Opportunities to meet each other *	Intrinsic fulfilment

* These values emerge as distinct within the higher education context

### Factors affecting the productivity of a research group

Areview conducted by Bland and Ruffin on the characteristics of a productive research environment outlines twelve factors that contribute to the development and leading of a productive research environment ( Bland, 1992), the factors outlined are:

1. Clear goals that serve a coordinating function
2. Research emphasis
3. Distinctive culture
4. Positive group climate
5. Assertive participative governance
6. Decentralised organisation
7. Frequent communication
8. Accessible resources, particularly human
9. Sufficient size, age, and diversity of the research group
10. Appropriate rewards
11. Concentration on recruitment and selection
12. Leadership with research expertise

We identified and explored these issues in the School of Pharmacy at UCLan. Within our school context, there were many part-time teacher practitioners undertaking novel and research-worthy teaching and assessment. When approached, the staff had the enthusiasm for pedagogical study but felt unable to do so due to lack of experience and time. Furthermore, laboratory-based research staff were also willing to participate but were unsure of the methods required and concerned about the time impact on their lab work ( Weller, 2011).

## Methods

### The goal of the new structure

Having explored the concerns previously described within the school, we set out to develop a collaborative research structure, incorporating as many of Bland and Ruffin’s factors and the values of a research community as possible. The structure needed to encompass the whole school and easily transferable to different settings. We also aimed to ensure the structure was easy to access, transparently designed, and effectively exploited all staff’s existing skills and knowledge. This led to the development of a collaborative, adhocracy research group called the Pedagogic Interest Group (PIG).

### The collaborative, adhocracy research group structure, PIG

**Figure 1:**
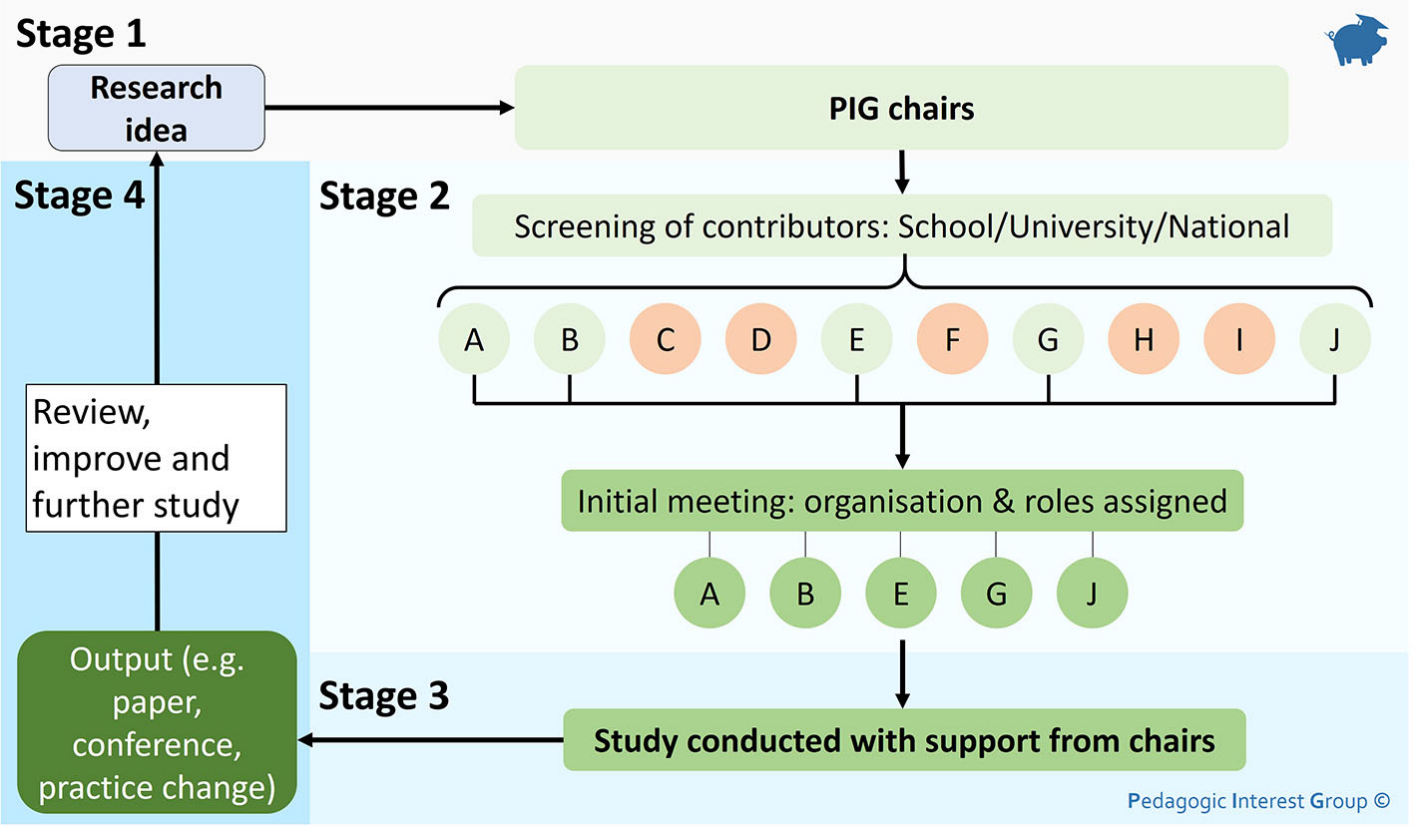
The typical process for running a project using the “collaborative adhocracy” structure of the Pedagogic Interest Group

Broadly the PIG structure is what we have termed an adhocracy, with bespoke members of staff from across the school, university and beyond, uniquely assembled for each project. Henry Mintzberg defined adhocracy as a “ *flexible structure that morphs to meet needs and where decisions are devolved, and coordination relies on good communication*” ( Mintzberg, 1989). The group is coordinated by two independent chairs who are research active in the area. PIG was initially implemented with a presentation at the beginning of 2020, where a clear direction and vision was set out (contributing to Bland and Ruffin’s first Factor). The whole school and faculty research staff were invited (with a session recording made available) so to reach as wide an audience as possible, working towards Factor 8, a large and diverse group. The summary process for how a project runs through PIG is outlined in Figure 1.

Lettered circles represent a pool of people who would be screened to contribute to a project

### Stage 1

Initially, in **Stage 1** of, a staff member who would like to run a project, approaches one of the two chairs to discuss the project and contact all potentially relevant staff to be involved. Therefore, it is essential to keep an up-to-date database of staff and their expertise, facilitating information sharing and collaborative research. The chairs curate this, and as such, the chair’s impartiality is of concern. For this reason, a minimum of two independent chairs is key, facilitating multiple people who can be approached with project ideas and to act as a second impartial mediator if required.

### Stage 2

One of the chairs will then set up **Stage 2**, assembling all interested staff for an initial meeting where the project and initial research question is defined, with roles and responsibilities agreed, meeting Factor 1, 11 and contributing to Factor 7, setting clear goals. At the start, defining roles has proved a useful step in the process, with projects running efficiently and no mediation being required from chairs in any projects so far, overall saving time and energy. Potential outputs are also discussed at this meeting to inform **Stage 4** of the process to foster tangible returns and drive research. If no strong output can be identified, be that internal or external, the value of the project is re-assessed, contributing to factor 10, ensuring appropriate reward.

### Stage 3

Most of the time is then spent on **Stage 3**, the duration of which is project dependent. During this step, even if they are not directly involved, the chairs are available for consultation and mediation, providing a sounding board, further contributing to factor 7. If further help is needed, additional staff members may be brought in as required for support and guidance.

### Stage 4

Once a project is finished, **Stage 4** ensures that the desired output is achieved as agreed in **Stage 2**. The project is reviewed and documented; so that the list of staff and their experience/expertise is kept up to date. Then, a comprehensive list of outputs associated with PIG is kept. This can be used for future reference in project involvement, allowing cross-pollination of ideas, developing new projects and funding applications.

## Results

In less than one year from the development and introduction of the PIG structure at UCLan, seven independent pedagogic research projects have been started, with two papers published, one under review, two in preparation, one abstract accepted at an international conference and a further fifteen funded undergraduate research projects completed ( Lunn *et al.*, 2020; Lunn, Cogan and Manfrin, 2021).

The adhocracy structure of PIG makes it easy to adapt to new situations, different institutions or research areas. In the first year of the structure, three other UK universities and four distinct disciplines (pharmacy, biomedical sciences, computer science and linguistics) have been successfully incorporated into PIG projects. The staff were included as collaborators in projects focusing on improving student learning in laboratory and lecture settings. Their easy inclusion into these projects increased the reachable student population and, therefore, the sample size four-fold and shows the flexible nature of the structure. These people (having agreed) will now be known to PIG and can be involved in research projects run by anyone in the wider group without having previously known them.

## Implications for practice

The collaborative adhocracy structure described allows all staff in a department to be research active, facilitating collaboration in an equitable and transparent way. If a researcher needs specific help (expertise or time), the PIG structure allows collaborators to be found even if the researcher doesn’t personally know anyone. This builds into the PIG structure the values of alternative perspectives, networking, having multiple sources of ideas, overcoming intellectual isolation and collaboration. By allowing the full inclusion of part-time staff such as teacher practitioners, research is enhanced by incorporating the knowledge of current trends and good practice from the workplace (particularly in medical fields). This keeps research and teaching relevant to the workplace, enhancing the student experience.

The flexible nature of the structure means that research projects can be run across a range of disciplines without impacting too heavily on one researcher. However, this approach requires a chairperson to arrange the initial meetings in a project, which can be time-consuming. A minimum of two chairs is required for impartiality and a fair spread of the workload. This also meets” Dunn’s Factor 5, minimising the centralisation of management. By following the structure for a project as described in Figure 1, clear goals are set and agreed on by all members, incorporating Factor 2, 4 and 6, with all members being included and useful.

However, creating a new working group for each project could generate the risk of losing a sense of belonging and identity, which therefore, must be fostered by the chair-people. The PIG structure, whilst widening participation and inclusion of alternative perspectives, requires open and honest conversations in the initial meeting so that the correct and motivated staff are included in each project to meet Factor 11, selecting the best people for a project.

## Conclusions

The PIG’s structure is based on a collaborative adhocracy. PIG provides a framework for incorporating collaborative pedagogical research into existing higher education research structures, fairly and flexibly taking advantage of existing skills and resources. The structure’s success relies on staff participation with two chairs providing a clear group vision and identity.

## Take Home Messages

• Pedagogic research can often be overlooked or be of poor quality due to existing research structures in universities
• A high-quality pedagogic research offering contributes to REF, TEF and student learning and satisfaction
• Implementation of a new flexible, collaborative adhocracy structure called the Pedagogic Research Group (PIG) has been shown to help improve the pedagogic research output at a UK pharmacy school

## Notes On Contributors

Dr. Andrew Martin Lunn is a lecturer in pharmacy and pharmaceutics at the University of Central Lancashire (UCLan), UK. ORCiD: https://orcid.org/0000-0003-2884-2755

Dr. Andrea Manfrin is the faculty director of research and chair professor of pharmacy practice at the University of Central Lancashire (UCLan), UK. ORCiD: https://orcid.org/0000-0003-3457-9981
