# Pedagogic Interest Group: a novel and proven collaborative, adhocracy research group structure

**DOI:** 10.15694/mep.2021.000061.2

**Published:** 2021-09-03

**Authors:** Andrew Martin Lunn, Andrea Manfrin

**Affiliations:** 1 University of Central Lancashire; 2 University of Central Lancashire

**Keywords:** Education, Management, Pedagogy, Research, Strategy, Structure

## Abstract

This article was migrated. The article was marked as recommended.

**Background:** Teaching is a core activity for universities, and pedagogic research is essential for improving student experience, staff satisfaction, and research and teaching quality. Pedagogic research is often performed as a secondary research area or by part-time staff, requiring good collaboration. Existing research structures in universities often result in pedagogic research falling through the gaps and for quality work and pedagogic improvements to be missed.

**Aim:** The aim was to develop a clear and flexible structure to improve participation and output of pedagogic research in the School of Pharmacy and Biomedical Sciences at the University of Central Lancashire.

**Method:** A collaborative adhocracy called the Pedagogic Interest Group (PIG) was created in January 2020. It was designed to allow collaborative, flexible research projects to be easily set up by any staff member. The group supervises and organises a bespoke team of people for each project, drawing on all previously involved staff’s expertise and contacts through an initial project meeting organised by an independent group chair. Each project group runs independently, with further help available from the group chairs.

**Results:** Under the PIG structure, seven projects have been undertaken in less than one year. Two papers were published, one under review, two in preparation, one abstract accepted at an international conference, and fifteen funded undergraduate research projects completed. Part-time teaching staff are more involved in the research. Internally, three departments and externally, three other UK universities have been collaboratively involved in research projects.

**Conclusion:** The PIG structure works and depends on staff’s continued engagement and at least two independent chairs for impartiality and transparency.

## Introduction

### Background

Pedagogic research represents the theoretical and/or conceptual understanding of teaching and learning processes, experiences and outcomes. It is conducted in many university departments and is a critical part of academic life, informing and improving the university’s core function of teaching; simultaneously, it contributes to the research output of a department. In the UK such teaching and research quality is measured and evaluated by the Research Excellence Framework (REF) and Teaching Excellence Framework (TEF) ( Swing and Ross, 2016; Gunn, 2018; Morris, 2020). As such, pedagogical research can contribute significantly to the success of a department and university. In our experience, pedagogic research may not be the primary research focus of academics or may be conducted by part-time teacher practitioners. This is the case in the School of Pharmacy and Biomedical Sciences at the University of Central Lancashire (UCLan); therefore, a new pedagogical research structure was devised and implemented, called the Pedagogic Interest Group (PIG).

### Traditional research structure(s)

The traditional research structure at a University is often organised in a subject-specific hierarchical manner, with individual researchers pursuing their area of expertise. Looking at the structure in a top-down approach, the university completes an audit of its research activity (REF in the UK), dividing funding up between faculties and departments. Each department of the university will then be further organised, potentially by discipline, with researchers working on their areas of expertise and hopefully collaborating within the department, university or broader research context, possibly in research communities ( Chirikov, 2013; Ng and Pemberton, 2013; Webber and Calderon, 2015). Researchers are, however, responsible for securing further funding and producing discipline-specific research outputs for the university as an individual. Such a competitive funding system that rewards individuals is potentially at odds with a truly collaborative culture ( Woiwode and Froese, 2020). Some structures, such as centres for excellence, have shown promise in increasing collaborative research between multiple disciplines ( Borlaug and Langfeldt, 2019). Nevertheless, there is often no clear structure or guidance on achieving a successful and collaborative research strategy, which can lead to bias in who collaborates, favouring those with pre-existing connections ( Birnholtz, 2007; Holman and Morandin, 2019).

### Pedagogic context

When this research landscape is applied to pedagogical research, particularly in a professional discipline such as pharmacy, medicine or nursing, further challenges are faced; for example, conducting research jointly within the governance of a university and hospital ( Engbers *et al.*, 2013; Cotton, Miller and Kneale, 2018). The research must also fulfil teaching quality criteria (TEF in the UK); many departmental staff will be teaching focussed, employed predominantly as experts in their field with little experience in producing research outputs (e.g. teacher practitioners) ( Laird, 2012). Furthermore, in clinical disciplines, patients who may have no academic background or awareness of pedagogic research are increasingly teaching as “experts by experience” ( Lunn *et al.*, 2020). For those who are unsure where to start, what constitutes a worthwhile research study or who struggle to find the time, a lack of institutional pedagogical research structure is a major barrier to entry ( Stierer and Antoniou, 2004; Cotton, Miller and Kneale, 2018).

### Research-Based Communities of Practice

Ng and Pemberton suggested that research is an integral part of the work in higher education institutions looking at the value of membership in communities of practice in higher education, and the potential impact on subsequent research ( Ng and Pemberton, 2013). Their research focused on:

• What individuals gain from their membership
• How such membership enhances their research
• Where and how communities of practice can integrate within higher education, and the role they play in developing research outputs

Communities of practice are formed naturally and informally, bound together by shared expertise ( Wenger and Snyder, 2000). Some examples of communities of practice are clinical practice, business management and information communication.

Ng and Pemberton identified 20 values of communities of practice; the values that emerge as distinct to a higher education context are:

• Alternative perspective and cross-pollination of ideas
• Time and energy-saving
• Overcoming intellectual isolation
• Fostering of tangible returns
• Driving research
• Synergy and leverage
• Opportunities to meet each other

### Factors affecting the productivity of a research group

A *review conducted by Bland and Ruffin on the characteristics of a productive research environment* outlines twelve factors that contribute to the development and leading of a productive research environment ( Bland & Ruffin, 1992). The factors outlined are:

1. Clear goals that serve a coordinating function,
2. Research emphasis
3. Distinctive culture
4. Positive group climate
5. Assertive participative governance
6. Decentralized organization
7. Frequent communication
8. Accessible resources, particularly human
9. Sufficient size, age, and diversity of the research group
10. Appropriate rewards
11. Concentration on recruitment and selection
12. Leadership with research expertise

We identified and explored these issues in the School of Pharmacy at the UCLan. Within our school context, there were many part-time teacher practitioners undertaking novel and research-worthy teaching and assessment. When approached, the staff had the enthusiasm for pedagogical study but felt unable to do so due to lack of experience and time. Furthermore, laboratory-based research staff were willing to participate but were unsure of the methods required and concerned about the time impact on their lab work ( Weller, 2011).

### Aim

To develop a clear and flexible structure to improve participation in and the output of pedagogic research in the School of Pharmacy and Biomedical Sciences at the University of Central Lancashire.

## Methods

### The goal of the new structure

Having explored the concerns previously described within the school, we set out to develop a collaborative research structure, incorporating as many of Bland and Ruffin’s factors and the values of a research community as possible. The structure needed to encompass the whole school and easily transfer to different settings. We also aimed to ensure the structure was easy to access, transparently designed, and effectively exploited all staff’s existing skills and knowledge. This led to developing a collaborative, adhocracy research group called the Pedagogic Interest Group (PIG).

### The collaborative, adhocracy research group structure, PIG

**Figure 1:**
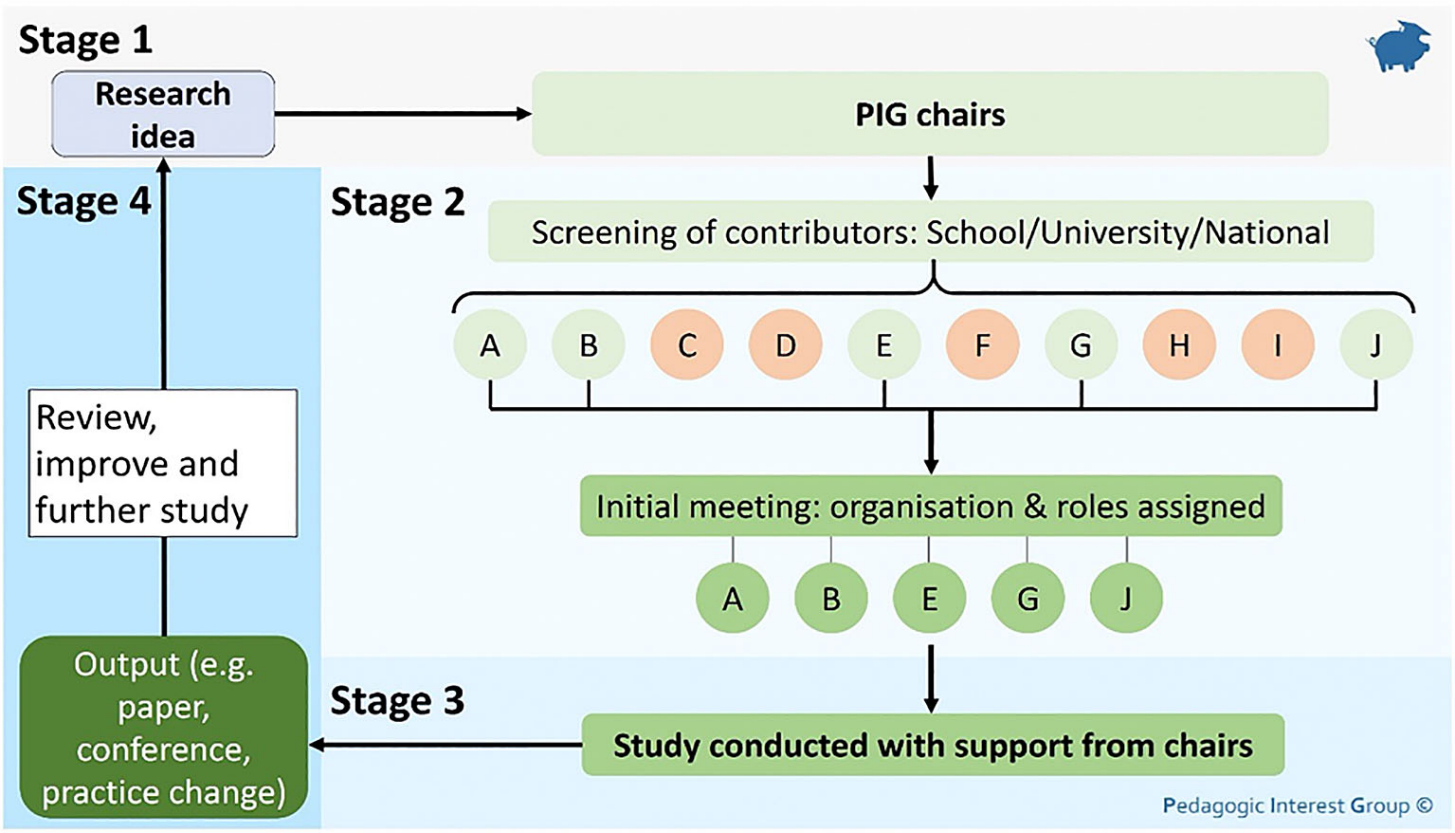
The typical process for running a project using the “collaborative adhocracy” structure of the Pedagogic Interest Group

Broadly the PIG structure is what we have termed an adhocracy, with bespoke members of staff from across the school, university and beyond, uniquely assembled for each project. Henry Mintzberg defined adhocracy as a “flexible structure that morphs to meet needs and where decisions are devolved, and coordination relies on good communication” ( Mintzberg, 1989). The group is coordinated by two independent chairs who are research active in the area. PIG was initially implemented with a presentation at the beginning of 2020, where a clear direction and vision was set out (contributing to Bland and Ruffin’s first Factor of clear goals). The whole school and faculty research staff were invited (with a session recording made available) so to reach as wide an audience as possible, working towards Factor 9, a large and diverse group. The summary process for how a project runs through PIG is outlined in Figure 1.

Lettered circles represent a pool of people who would be screened to contribute to a project

Each stage of the process is further explained below, and a real example of a completed project used to help explain the process.

### Stage 1

Initially, in **Stage 1**, a staff member who would like to run a project approaches one of the two chairs to discuss the project and contact all potentially relevant staff to be involved. Therefore, it is essential to keep an up-to-date database of staff and their expertise, facilitating information sharing and collaborative research. The chairs curate this, and as such, the chair’s impartiality is of concern. For this reason, a minimum of two independent chairs is key, facilitating multiple people who can be approached with project ideas and to act as a second impartial mediator if required.

Example stage 1: A staff member wanted to evaluate the impact of Patient As Teacher (PAT) classes on students. They had run the sessions for several years, but had no background in research, so after hearing about PIG, they approached one of the chair-people.

### Stage 2

One of the chairs will then set up **Stage 2**, assembling all interested staff for an initial meeting where the project and initial research question is defined, with roles and responsibilities agreed, meeting Factor 1, 11 (clear goals and a concentration on recruitment and selection) and contributing to Factor 7 (frequent communication), setting clear goals. The initial meeting has proved crucial; for some ideas it has meant no further time was spent on them, and for those that have continued, defining roles early has resulted in all projects running efficiently so far, overall saving time and energy. Potential outputs are also discussed at this meeting to inform **Stage 4** of the process to foster tangible returns and drive research. If no strong output can be identified, be that internal or external, the value of the project is re-assessed, contributing to factor 10, ensuring appropriate reward.

Example stage 2: An initial meeting was set up with all people known to have an interest or relevant expertise. This included staff from the school of pharmacy, the school of social work and a colleague with expertise in statistics. Four people were identified as interested and required for the project and were given the following roles:

Person 1: Study design, ethical approval, manuscript writing

Person 2: Study design, facilitating study in PAT classes

Person 3: Facilitating study in PAT classes, manuscript writing

Person 4: Statistical analysis plan, design, ethical approval.

To ensure correct ownership of the project, the chair facilitated the person whose idea it was to lead the meeting and have an ultimate say on how it was run. A minimum of a peer reviewed article was outlined as the outcome of this project, with the potential to lead to a larger scale study.

### Stage 3

Most of the time is then spent on **Stage 3**, the duration of which is project dependent. Even if they are not directly involved during this step, the chairs are available for consultation and mediation, providing a sounding board, further contributing to factor 7 (frequent communication). If further help is needed, additional staff members may be brought in as required for support and guidance.

Example stage 3: The study was designed to include quantitative and qualitative analysis (person 1, 2, 3, 4). As PIG had existing ethical approval, this study gained approval by a chair’s action from the ethics committee (person 1 and 4). A previously validated evaluation tool was identified, adapted, and distributed to students after PAT sessions via an online platform (All staff). The results were analysed and interpreted by person 4.

### Stage 4

Once a project is finished, **Stage 4** ensures that the desired output is achieved as agreed in **Stage 2**. The project is reviewed and documented; so that the list of staff and their experience/expertise is kept up to date. Then, a comprehensive list of outputs associated with PIG is kept. This can be used for future reference in project involvement, allowing cross-pollination of ideas, developing new projects and funding applications.

Example stage 4: After the analysis, a suitable journal was identified and person 1 drafted the manuscript. All staff involved then contributed to the review process (as would be normal) and publication was successful. One of the chairs made a record of the people involved, and techniques used, which led to a secondary outcome of a talk at a national conference. The curated list of projects was used to identify it as a suitable project for abstract submission. This record has subsequently yielded a second project evaluating a large event held at the university and facilitated access to patients to assess learning in cognitive impairment.

## Results

In less than one year from the development and introduction of the PIG structure at UCLan, seven independent pedagogic research projects have been started, with two papers published, one under review, two in preparation, one abstract accepted at an international conference and a further fifteen funded undergraduate research projects completed ( Lunn *et al.*, 2020; Lunn, Cogan and Manfrin, 2021).

The adhocracy structure of PIG makes it easy to adapt to new situations, different institutions, or research areas. In the first year of the structure, three other UK universities and four distinct disciplines (pharmacy, biomedical sciences, computer science and linguistics) have been successfully incorporated into PIG projects. The staff were included as collaborators in projects focusing on improving student learning in laboratory and lecture settings. Their easy inclusion into these projects increased the reachable student population; therefore, the sample size four-fold and shows the flexible nature of the structure. These people (having agreed), will now be known to PIG and can be involved in research projects run by anyone in the wider group without having previously known them.

## Implications for practice

The collaborative adhocracy structure described helps all staff to be research active, facilitating collaboration in an equitable and transparent way. If a researcher needs specific help (expertise or time), the PIG structure allows collaborators to be found even if the researcher doesn’t personally know anyone. This builds into the PIG structure the values of alternative perspectives, networking, having multiple sources of ideas, overcoming intellectual isolation and collaboration. By allowing the full inclusion of part-time staff such as teacher practitioners, research can be enhanced by incorporating the knowledge of current trends and good practice from the workplace (particularly in medical fields). This can help to keep research and teaching relevant to the workplace, enhancing the student experience.

It is the authors experience that the flexible nature of the structure has developed a distinctive and positive culture, achieving factors 3 and 4. This flexibility has allowed research projects to run across various disciplines, one real example between pharmacy, biosciences and linguistics. However, this approach requires a chairperson to curate projects and expertise to be shared, which can be time-consuming. Due to the workload and impartiality, we have found that a minimum of two chairs is required. This also meets Bland and Ruffin’s Factor 6, minimising the centralisation of management. By following the structure for a project as described in Figure 1, clear goals are set and agreed on by all members, incorporating Factors 1 and 5 (clear goals and participative governance), with all members being included and useful.

However, creating a new working group for each project could generate the risk of losing a sense of belonging and identity, which the chair-people must foster. While potentially widening participation and inclusion of alternative perspectives, the PIG structure requires open and honest conversations in the initial meeting so that the correct and motivated staff are included in each project to meet Factor 11, concentration on recruitment and selection.

The chairs gained their roles by default in the case presented, being the people who conceived and developed PIG. Our experience suggests that diversity is key. One senior and one more junior member of staff has kept PIG rooted and approachable but given it reach to the university management. It is likely that when a group like this is being developed, those who have driven the process will take on the roles of the chair. However, where selection may be required, we would tentatively suggest a de-facto chair to set up a group’s initial membership and explain the role, with volunteers for the chairs role then being sought. In the case of too many volunteers, we would hope peer discussion or a members’ vote could resolve this, with an annual review.

## Conclusions

The PIG structure is based on a collaborative adhocracy. PIG provides a potential framework for incorporating pedagogic research into existing higher education research structures, fairly and flexibly taking advantage of existing skills and resources. The structure’s success relies on staff participation with multiple chairs providing a clear group vision and identity.

## Take Home Messages

• Pedagogic research can often be overlooked in universities; however, high-quality pedagogic research can greatly contribute to research and teaching quality, and student learning and satisfaction
• A new flexible, collaborative adhocracy structure called Pedagogic Interest Group (PIG) was designed and implemented at a UK pharmacy school
• The new structure was designed to facilitate an inclusive and transparent path into pedagogic research and collaboration
• In under a year, the new structure has facilitated: seven staff projects, two published papers and fifteen undergraduate projects.

## Notes On Contributors

**Dr. Andrew Martin Lunn** is a lecturer in pharmacy at the University of Central Lancashire (UCLan), UK. ORCID: https://orcid.org/0000-0003-2884-2755

**Dr. Andrea Manfrin** is the faculty director of research and chair professor of pharmacy practice at the University of Central Lancashire (UCLan), UK. ORCID: https://orcid.org/0000-0003-3457-9981
