# Supplementary material for: Pedagogic Interest Group: a novel and proven collaborative, adhocracy research group structure
Source: MedEdPublish (2016). 2021 Sep 3;10:61. Originally published 2021 Mar 2. [Version 2] doi: 10.15694/mep.2021.000061.2 (PMC10939623; doi:10.15694/mep.2021.000061.2)
Supplement: Peer review history

# Reviewer report: Reviewer response for version 2

**DOI:** 10.21956/mep.20216.r31303

**Published:** 2021-09-07

**Authors:** Megan Anakin

**Affiliations:** 1 University of Otago

This review has been migrated. The reviewer awarded 4 stars out of 5 Thank you for publishing this revised version of your article. Thank you for replacing the REF and TEF abbreviation in the abstract with “research and teaching quality”. The background section of this practical tip article has been revised to enable the UK context and references to be understood by an international reader. Thank you for replacing Table 1 with a list, as suggested by reviewer Ken Masters, however an explanation is missing about how these values were used to develop the Pedagogic Interest Group. Likewise, the 12 factors listed from Bland & Ruffin require explanation as to how they were used to inform the development of the Pedagogic Interest Group.Thank you for including an example to illustrate a research idea and processes of the group that occurred in stages 1, 2, 3, and 4. The implications for practice section could be further strengthened by making references to the literature to make comparisons with other initiatives that seek to improve participation in educational research studies and outputs. The Take Home Messages now relate directly to the aim.

Reviewer Expertise:

NA

No decision status is available

# Reviewer report: Reviewer response for version 2

**DOI:** 10.21956/mep.20216.r31302

**Published:** 2021-09-06

**Authors:** Ken Masters

**Affiliations:** 1 Sultan Qaboos University

This review has been migrated. The reviewer awarded 4 stars out of 5 The authors have done well to address my main concerns with Version 1 of the paper, and have produced a much-improved Version 2. Although the experience outlined is strongly grounded in local specifics, there are many elements and principles that would be more broadly applicable, and so the paper will be of value to others.

Reviewer Expertise:

NA

No decision status is available

# Reviewer report: Reviewer response for version 1

**DOI:** 10.21956/mep.19149.r27692

**Published:** 2021-06-08

**Authors:** Ken Masters

**Affiliations:** 1 Sultan Qaboos University

This review has been migrated. The reviewer awarded 2 stars out of 5 An interesting paper on the creation of a Pedagogic Interest Group focusing on medical education research.The project described is useful. There are, however, several issues in the paper that need to be addressed:There is, unfortunately, a serious problem with Table 1. The lead-in to the table is “Lai and Pemberton (Ng and Pemberton, 2013) identified 20 values of communities of practice, which are summarised in Table 1.” This gives the impression that Ng and Pemberton identified these items, and the authors of the current paper have summarised Ng and Pemberton’s work into this table. This is misleading:• Table 1 is almost a direct replication of Ng and Pemberton’s Table 4 (p.1530).• The differences are minor (e.g. removal of Ng and Pemberton’s numbers, removal/insertion of a few words)In addition, some of the alterations have led to errors or differences not explained in the text.As a result, for Version 2 of the paper, I would recommend that the authors do ONE of the following:• either remove their Table 1 entirely, and refer only to the items in Ng and Pemberton’s Table 4, or• reproduce Ng and Pemberton’s Table 4 as is, making clear that this is their table. In order to do so, though, the authors would need to obtain permission from the copyright holders, and would need to declare that permission in the Acknowledgments. If the authors then wish to indicate that they have worked off a slightly modified version of Ng and Pemberton’s Table 4, they should then describe the modifications, and why these were necessary.It is really for this reason that I have awarded a low rating to the current version of the paper, as this is a rather serious oversight.Some other issue:• “and REF and TEF scores” In the Abstract, these require expansion. As the journal is aimed at an international audience, these require some further explanation in the body of the paper, where they are first mentioned.• As much of the paper refers to these items, and they appear to be the strong motivation for the formation of the group, the paper should clarify that it is aimed particularly at this (national or regional?) requirement, and so may not be more widely applicable.• “Pedagogic research is often not the primary research focus of academics or is conducted by part-time teacher practitioners.” This may be the authors’ experience, but does require support from the literature if it is to be taken as a widely-applicable statement.• “Lai and Pemberton (Ng and Pemberton, 2013) identified 20 values….” There appears to be an error in this citation. See also the caption for Table 1.• Bland 1992: The citation (and the reference) gives only Bland; there are two authors of that article: Bland and Ruffin (as is mentioned later in the text). Please correct this citation and reference.• “This also meets ' 'Dunn's Factor 5….” The reference to “'Dunn's Factor 5” is not clear, and the punctuation in this sentence appears to be incorrect.• “in each project to meet Factor 11, selecting the best people for a project” It would be best to use the wording of the original, rather than an interpretation of the original wording.• There are a few other minor language and punctuation errors in the paper that need to be corrected.I look forward to Version 2 of the paper in which these issues are addressed.

Reviewer Expertise:

NA

No decision status is available

# Reviewer report: Reviewer response for version 1

**DOI:** 10.21956/mep.19149.r27691

**Published:** 2021-04-18

**Authors:** Megan Anakin

**Affiliations:** 1 University of Otago

This review has been migrated. The reviewer awarded 3 stars out of 5 Thank you for presenting your article on a collaborative research group structure. We are trying to set up support structures to foster health professions education research at our own institution and agree that this Pedagogic Interest Group (PIG) has merit as signposted in the abstract of the article. We are keen to share this publication with colleagues as it purports a number of positive group research outputs. Our suggestions are intended to enhance the presentation of your ideas. Please spell out abbreviations REF and TEF in the abstract because an international reader may not know what they are. Alternatively, please consider using generic terms (measurements of teaching quality) in the abstract and explain national frameworks such as REF and TEF in the background to the article. In the introduction section on pedagogic context, the statement “some of the teachers may even increasingly be patients” is confusing. Please re-word to show that this means patients as educators and explain why this has been framed as a challenge. Please reconsider the usefulness of the research based communities of practice table and list to the reader by better integrating them into the paper. Consider presenting Table 1, as a list or figure (see guidelines such as APA). Please consider integrating and synthesizing ideas from the literature into relevant examples of how you applied these values and frameworks in practice. Consider naming the factors to add clarity. It would be helpful to explain why funding is at odds with collaborative culture. To enable us to better recreate this structure in our own institution demonstrating wider applicability to practice, please consider providing further support statements about the initiative with references and examples from practice. By stating something is so, does not make it so. For example, explain how the bespoke chairpersons were chosen or how the group was facilitated. With respect to academic writing, please omit the word ‘etc.’ from the article to make the sentence formal and complete. Please correct referencing errors such as: Ng and Pemberton (referred to in the text as Lai and Pemberton); Dunn (is this Bland & Ruffin?). In the results section, please move citations next to papers published. Please avoid definitive statements (“will”) unless you support them with evidence. Please also consider revising statements, which use words like “often”, “frequent”, “seldom”, with more specific terms and support your claims with examples or references.  Please state the aim in the body of the article. Please consider revising the take home messages so that they relate directly to the aim and findings of the project. Finally, if we were to use this model in our own institution, we do not feel that the abbreviation PIG would be helpful to recruit members. We would be very happy to review a revised version of this article.

Reviewer Expertise:

NA

No decision status is available
